# HLA‐Knockout: Enabling Allele‐Specific Knockout of HLA Class I Genes for Immunogenic Engineering

**DOI:** 10.1111/tan.70548

**Published:** 2026-01-21

**Authors:** Connor Mattivi, Shiyu Wang, Longtao Ji, Qian Xiao, Jian Cao

**Affiliations:** ^1^ Rutgers Cancer Institute, Rutgers, the State University of New Jersey New Brunswick New Jersey USA; ^2^ Institute of Modern Biology, Nanjing University Nanjing China; ^3^ The Affiliated Drum Tower Hospital of Nanjing University Medical School, Nanjing University Nanjing China; ^4^ Department of Medicine Robert Wood Johnson Medical School, Rutgers, the State University of New Jersey New Brunswick New Jersey USA

## Abstract

The interaction between T‐cell receptors (TCRs) and antigenic peptides presented by HLA molecules is fundamental to adaptive immunity. However, the extreme polymorphism of HLA genes poses major challenges for transplantation, antigen discovery, immunotherapy and studies of allele‐specific function. Although CRISPR/Cas9 has transformed gene editing, existing sgRNA design tools are not optimised for knockout of HLA Class I genes due to their high rates of polymorphism. To address this, we developed HLA‐Knockout (https://hlaknockout.rutgers.edu), a novel web‐based tool that enables precise, allele‐specific targeting of HLA Class I genes. HLA‐Knockout retrieves user‐defined HLA sequences from the IPD‐IMGT/HLA database and applies stringent design criteria, including mismatch filtering and PAM disruption analysis, to ensure high specificity and minimal off‐target effects on non‐target HLA Class I alleles. Using HLA‐Knockout, we achieved efficient single‐ and double‐allele HLA Class I knockouts in human cells without disrupting non‐target HLA Class I alleles. Functional assays confirmed allele‐specific loss of antigen‐specific TCR activation, validating the platform's utility. HLA‐Knockout provides a unique resource for dissecting HLA‐restricted immune interactions and has broad applications in transplantation biology, autoimmunity and cancer immunotherapy.

## 1 Background

The human adaptive immune system relies on the recognition of non‐self‐antigens by B and T lymphocytes through their respective surface receptors [1]. For T cells, antigen recognition is mediated by T‐cell receptors (TCRs), which recognise peptides presented by major histocompatibility complex (MHC) molecules, known in humans as HLAs, on the surface of antigen‐presenting cells (APCs) [2]. HLA genes span approximately 3.6 Mb on the short arm of chromosome 6 and are divided into three classes based on function [3]. Class I HLA genes (HLA‐A, HLA‐B and HLA‐C) encode molecules expressed on nearly all nucleated cells and present intracellular peptides to CD8+ T cells [4].

HLA genes are among the most polymorphic loci in the human genome. The IPD‐IMGT/HLA database has documented over 41,000 unique HLA alleles, including more than 28,000 for Class I genes alone [5]. This diversity enhances immune recognition at the population level but presents challenges for applications such as organ transplantation and TCR‐based immunotherapy. For example, matching donor‐recipient HLA alleles remains a critical barrier in transplantation [6], and selective knockout of HLA alleles is being explored to create hypoimmunogenic iPSCs [7]. In immunotherapy research, determining the HLA restriction of antigen‐specific TCRs often requires expression of single HLA alleles in HLA null backgrounds [8]. Alternatively, knockout of individual HLA alleles in APCs can be used to map TCR restriction. This approach is particularly valuable when the cognate antigen is unknown, since it cannot be overexpressed in HLA‐null cells for functional testing.

CRISPR/Cas9 enables targeted gene editing through the introduction of double‐strand breaks (DSBs) by a Cas9 endonuclease guided by a 20‐nt single guide RNA (sgRNA). In mammalian cells, DSBs are typically repaired via error‐prone non‐homologous end joining, often resulting in gene disruption [9, 10]. While many tools exist for sgRNA design [11, 12, 13, 14], none are tailored for allele‐specific targeting of HLA genes, which requires precision to discriminate between highly similar alleles.

To address this gap, we have developed a web‐based tool specifically designed to identify guide sequences for targeting human HLA Class I genes with allele‐level specificity. This tool scans the desired allele for candidate guide sequences and filters out those with off‐target activity against non‐intended HLA alleles, using criteria such as PAM sequence alterations and high numbers of mismatches within the target sequence. Guide sequences identified using this tool successfully achieved allele‐specific knockout of HLA Class I alleles in a human cell line and effectively abolished HLA allele‐specific T‐cell recognition. These results demonstrate the utility of this tool for applications in TCR‐based immunology and HLA‐restricted immune modulation studies.

## 2 Materials and Methods

### 2.1 Target Sequence Identification

User‐entered HLA allele names are used to retrieve corresponding information from the IPD‐IMGT/HLA Database, including the allele accession number, coding sequence and genomic sequence. The data generated in this study was based on IPD‐IMGT/HLA release version 3.60. If a partial allele name is entered (e.g., A*01:01), and multiple complete sequences exist (e.g., A*01:01:01 or A*01:01:02), the tool will automatically autocomplete the input to the first full match and prompt the user for confirmation. If an entered allele is missing from the database or lacks associated sequence information, a warning is displayed to inform the user that these alleles cannot be used for target sequence generation or off‐target filtering. Once allele identity has been confirmed and sequence availability has been verified, the user is then prompted to select one or more alleles desired for knockout. Candidate target sequences are then identified from the genomic sequence of the selected allele(s) on both the forward and reverse strands. Each candidate consists of a 23‐nucleotide sequence, including the *Streptococcus pyogenes* Cas9 specific protospacer adjacent motif (PAM) NGG. All potential target sites within the selected knockout alleles are recorded. If multiple alleles are selected for knockout, only those target sequences that are shared across all selected alleles are retained. To ensure that Cas9 cleavage occurs in a coding region, each guide sequence is further filtered so that the cut site, located between the 17th and 18th nucleotide of the guide, falls within an exon or within 3 nucleotides of an exon boundary (5′ or 3′). This is determined by aligning the coding and genomic sequences of the selected alleles and mapping the candidate target sequences accordingly.

### 2.2 Off‐Target Filtration

Once a list of exon‐targeting candidate sequences has been generated, these candidates undergo an additional filtration step to ensure specificity for the intended knockout allele(s). Each candidate sequence is compared against the genomic sequences of all non‐targeted HLA Class I alleles using string matching with mismatch tolerance. Potential off‐target sites are aligned to the candidate guide sequence and evaluated for their likelihood of being cleaved by Cas9. Off‐target cleavage is predicted when both of the following conditions are met: (1) the off‐target site contains a functional PAM sequence (either NGG or AGA, regardless of the PAM found in the target allele); and (2) the off‐target site exhibits high sequence homology to the guide sequence. The filtering process begins by checking the PAM site. If the PAM is not NGG or AGA, the off‐target site is considered non‐cleavable by Cas9, and the candidate guide is retained. If the PAM is NGG or AGA, suggesting potential for Cas9 cleavage, the candidate is subjected to further evaluation based on sequence similarity. Specifically, the 20‐nucleotide guide region (excluding the PAM) is aligned to the corresponding region in the off‐target allele. Each nucleotide mismatch is weighted based on its position, with greater weight assigned to matches proximal to the PAM. The resulting score reflects the possibility of editing, where lower scores indicate greater off‐target risk. Candidate sequences predicted to direct Cas9 cleavage in any non‐targeted allele are excluded. Only those guides with non‐functional PAMs or sufficient mismatches across all off‐target alleles are retained. The final output is a list of sgRNA candidates predicted to be highly specific for the selected HLA allele(s), with minimal risk of cleaving other alleles due to PAM disruption or low sequence homology.

### 2.3 Simulation of Target Sequence Identification Capability

Cancer cell line HLA typing information was retrieved from the TRON Cell Line Portal (TCLP) [14]. Cell lines with six unique HLA Class I alleles, where each allele had both genomic and coding sequences available in the IPD‐IMGT/HLA database, were selected to test the HLA‐Knockout algorithm (*n* = 432). For each cell line, we applied the HLA‐Knockout algorithm to identify candidate guide sequences for each allele independently (single‐allele knockout), as well as for both alleles of each gene locus (pan‐HLA‐A, pan‐HLA‐B and pan‐HLA‐C), which represent the most common intended use cases. The number of viable, allele‐specific target sequences identified for each allele or allele pair was recorded and subsequently plotted to visualise tool performance across diverse allele combinations.

### 2.4 Cloning and Cell Culture

293T cells and SK‐RC‐01 cells were cultured in DMEM and RPMI‐1640 medium, respectively, supplemented with 10% fetal bovine serum (FBS) and penicillin–streptomycin. DNA oligonucleotides for guide generation were obtained from Millipore Sigma. Guide sequences were cloned into the LentiCRISPRv2 vector (Addgene #52961) to generate stable knockout cell lines, following the procedure described previously [15]. Briefly, oligos were phosphorylated using T4 Polynucleotide Kinase (NEB M0201S) and annealed, then ligated into Esp3I‐digested LentiCRISPRv2 using T4 DNA Ligase (NEB M0202S). Ligation products were transformed into Stbl3 competent cells (Thermo Fisher C737303) and amplified using the Promega PureYield Plasmid Miniprep System (Promega A1222). All constructs were verified by Sanger sequencing (Azenta Life Sciences). Guide sequences used are: Ctrl, ATTGAGAATTCGTTTCAAGG; B2M, AGTCACATGGTTCACACGGC; A*02:01 sgRNA1, GAGGGTCCGGAGTATTGGGA; A*02:01 sgRNA2, CGCCTCCCACTTGTGCTTGG; A*03:01 sgRNA1, TCTCACCTTTACAAGCTGTG; A*03:01 sgRNA2, CGTGTCGTCCACGTAGCCCA; A*03:01 sgRNA3, CTCACCTTTACAAGCTGTGA; A*03:01 sgRNA4, GGCGCTTCCTCCGCGGGTAC; Pan‐A sgRNA1, GACGCCGAGGATGGCCGTCA; Pan‐A sgRNA2, TGGAACCTTCCAGAAGTGGG; Pan‐A sgRNA3, TTCTCCCCAGACGCCGAGGA; Pan‐A sgRNA4, CTGGTTGTAGTAGCCGCGCA; Pan‐C sgRNA1, GCGACGCCGCGAGTCCGAGA; Pan‐C sgRNA2, TGGCCTGGTCTCCACAAGCT; Pan‐C sgRNA3, AGGGGAGCCGCGGGCGCCGT; Pan‐C sgRNA4, CGTTGCTGGCCTGGCTGTCC. To generate lentivirus, 293T cells in 12‐well plates were transfected using Lipofectamine 2000 (Invitrogen) following the manufacturer's protocol. For each well, 750 ng of the lentiviral plasmid, 500 ng of psPAX2 and 250 ng of pMD2.G were used. After 48 h, virus‐containing supernatant was harvested, filtered through a 0.45 μm filter, and used to infect SK‐RC‐01 cells for 24 h. Infected cells were selected with puromycin for 4 days to eliminate untransduced cells.

### 2.5 Flow Cytometry

Cells were washed once with FACS staining buffer (PBS supplemented with 2% FBS and 2 mM EDTA) prior to surface staining with fluorophore‐conjugated antibodies. Antibodies were diluted in FACS buffer according to the manufacturers' instructions. Cells were resuspended in 100 μL of antibody solution and incubated at 4°C for 20 min in the dark. After staining, cells were washed twice with FACS buffer. Flow cytometric data were acquired on a FongCyte 3‐laser flow cytometer and analysed using FlowJo v10.6.2 (BD Biosciences). For detection of T‐cell transduction efficiency, PE anti‐mouse TCR β chain antibody (clone H57‐597, BioLegend, Cat# 109208) was used. HLA expression was assessed with the following antibodies: PE anti‐human HLA‐A,B,C (clone W6/32, BioLegend, Cat# 311406), PE anti‐human HLA‐A2 (clone BB7.2, BioLegend, Cat# 343306), Alexa Fluor 647 anti‐human HLA‐C (clone DT9, BioLegend, Cat# 373308), PE anti‐human HLA‐A3 (clone GAP.A3, Thermo Fisher, Cat# 12‐5754‐42).

### 2.6 TCR‐T‐Cell Generation

Non‐tissue culture‐treated 24‐well plates were coated overnight at 4°C with anti‐human CD3 and CD28 antibodies (BioLegend, 1 μg/mL in PBS, 0.5 mL/well). Human PBMCs were thawed, counted and seeded the next day at 6–8 × 10^6^ cells per well in 2 mL T‐cell medium (X‐vivo with 10% FBS, 1% penicillin–streptomycin, 2 mM L‐glutamine, 25 mM HEPES and 300 IU/mL IL‐2). For transduction, plates were coated overnight with retronectin (Takara, 10 μg/mL in PBS). Viral supernatant was added, and plates were spinoculated at 2000 × g for 1.5 h at 32°C. After removing supernatant, 0.25 × 10^6^ activated T cells in 1 mL medium were added and centrifuged again at 1500 rpm for 10 min. The following day, cells were transferred to tissue culture‐treated plates. Transduction efficiency was assessed on Day 6. Retrovirus was produced by transfecting 293T cells with retroviral plasmid (1.31 μg), RD114 (0.42 μg) and gag/pol (0.92 μg) using Lipofectamine 2000 (Invitrogen). Viral supernatant was collected 48 h later for use in transduction.

### 2.7 Co‐Culture

On Day 12 after PBMC activation, 2.5 × 10^4^ TCR‐T cells were co‐cultured with 2.5 × 10^4^ tumour cells in 200 μL T‐cell medium without IL‐2 supplementation in 96‐well flat‐bottom plates. Each condition was set up in triplicate. After 24 h, culture supernatants were collected and assayed for IFN‐γ concentration by ELISA according to the manufacturer's instructions (BioLegend).

## 3 Results

### 3.1 HLA‐Knockout Web Tool

**FIGURE 1:**
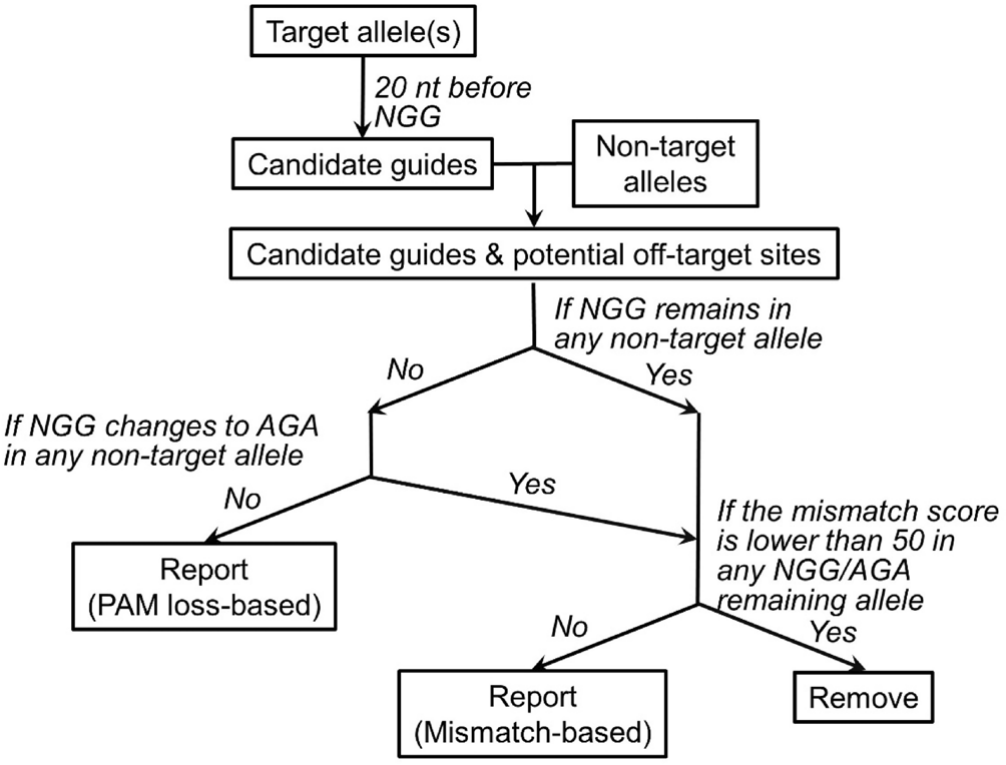
Workflow of HLA‐Knockout. The user inputs target and non‐target HLA alleles. HLA‐Knockout outputs selected guide sequences in two categories: (1) PAM loss–based: Allele‐specific guides due to PAM mutations in all non‐target alleles; and (2) Mismatch‐based: Allele‐specific guides due to sequence mismatches, without PAM disruption in at least one non‐target allele.

**FIGURE 2:**
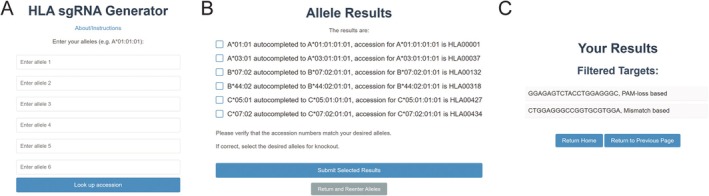
User interfaces of the HLA‐Knockout web tool. (A) Input panel for specifying HLA alleles. (B) Selection of target allele(s) for allele‐specific knockout. (C) Output display showing categorised guide sequences based on PAM loss or sequence mismatches.

The HLA‐Knockout website (https://hlaknockout.rutgers.edu) provides a streamlined workflow for generating sgRNAs that specifically target HLA alleles in a patient‐specific context (Figure 1). Users begin by entering up to six Class I HLA allele designations, following the standard nomenclature established by the WHO Nomenclature Committee for Factors of the HLA System [16] (Figure 2A). Allele names with fewer than four fields (e.g., HLA‐A*01) are accepted; however, this may result in multiple potential matches in the database. In such cases, the tool automatically selects the first complete allele name (e.g., HLA‐A*01:01:01:01) corresponding to the input and prompts the user to confirm the selection. Once verified, allele‐specific coding and genomic sequences are retrieved from the IPD‐IMGT/HLA Database [5]. Users are then prompted to select one or more alleles as knockout targets (Figure 2B). HLA‐Knockout proceeds to identify candidate guide sequences within the selected allele(s), filters out any guides that also target at least one non‐selected allele, and provides a final list of guide sequences predicted to achieve allele‐specific knockout (Figure 2C). Guide sequences determined to be allele‐specific due to PAM mutations in all non‐targeted alleles will be marked as ‘PAM based’, as these mutations guarantee no binding at the off‐target sites. Other guides, which contain significant mismatches in the guide sequence but retain intact PAMs in at least one non‐targeted allele, will also be provided. However, these may still carry a low risk of off‐target editing at one or more non‐targeted alleles.

### 3.2 Simulation Analyses

**FIGURE 3:**
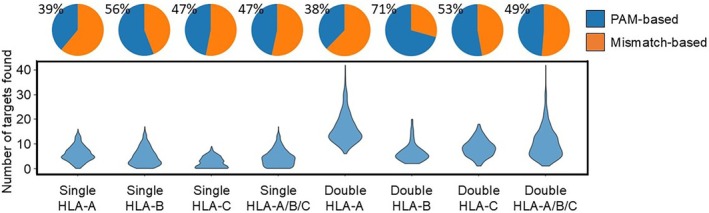
Simulation‐based evaluation of HLA‐Knockout. Number of allele‐specific sgRNAs identified for single‐allele knockout of HLA‐A, HLA‐B, HLA‐C, any HLA single Class I allele, double‐allele knockout (pan‐knockout) of HLA‐A, HLA‐B, HLA‐C or any HLA Class I gene based on 432 cell lines from the TCLP database. Pie charts indicate the proportion of PAM‐based and mismatch‐based guide sequences from the total number of guide sequences identified per category.

To assess the potential utility of the HLA‐Knockout tool, we obtained HLA Class I typing information from 432 cell lines in the TCLP database [17], each carrying six distinct HLA Class I alleles, two each for HLA‐A, HLA‐B and HLA‐C. For each cell line, we performed sgRNA design for (1) single‐allele knockout and (2) double‐allele knockout targeting both alleles of each HLA gene (HLA‐A, HLA‐B, or HLA‐C). For single‐allele knockouts, at least one viable guide sequence was identified in over 89% of cases (2329 out of 2592 attempts; Figure 3). These single‐allele knockout runs identified a total of 11,428 viable guide sequences, with an average of 4.43 guide sequences per run. For double‐allele knockouts, at least one shared guide sequence targeting both alleles was found in 100% of cases (1296 out of 1296 attempts; Figure 3). The double‐allele knockout runs identified a total of 13,489 viable guide sequences, with an average of 10.41 guide sequences per run. These results highlight the broad applicability and robustness of allele‐specific HLA knockout across diverse HLA genotypes.

### 3.3 Verification of Allele Specific Knockout

**FIGURE 4:**
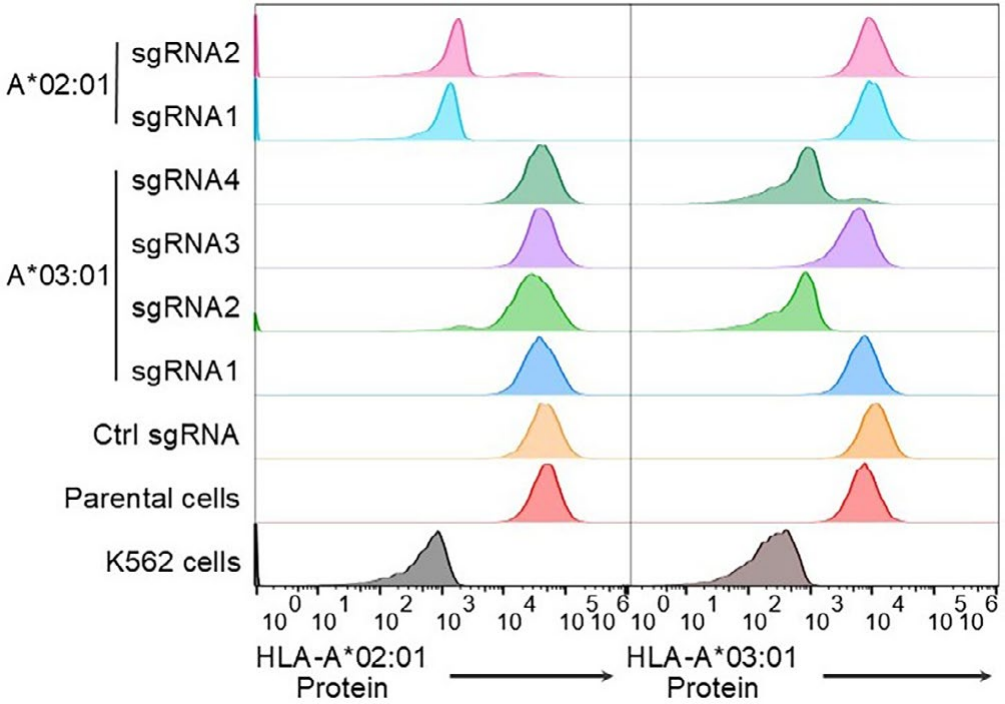
Validation of guide selection for allele‐specific HLA knockout. Flow cytometry analysis of HLA‐A*02:01 and A*03:01 protein expression in SK‐RC‐01 cells transduced with spCas9 and either a control sgRNA, two independent sgRNAs targeting HLA‐A*02:01, or four independent sgRNAs targeting HLA‐A*03:01. Parental SK‐RC‐01 cells and K562 cells were included as positive and negative controls, respectively.

**FIGURE 5:**
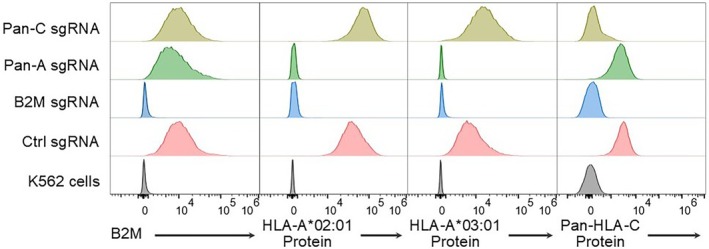
Validation of guide selection for HLA Class I gene‐specific knockout. Flow cytometry analysis of B2M (indicating total HLA Class I), HLA‐A*02:01 protein, HLA‐A*03:01 protein and pan‐HLA‐C protein expression in SK‐RC‐01 cells transduced with Cas9 and either a control sgRNA or an sgRNA targeting HLA‐A or HLA‐C. K562 cells were included as a negative control.

We then validated our approach using SK‐RC‐01 cells, which carry the following HLA alleles: HLA‐A*02:01, A*03:01, B*51:01, B*56:01, C*07:02 and C*14:02. HLA‐Knockout identified four A*02:01‐specific guide sequences and seven A*03:01‐specific guide sequences. It also identified 15 and 9 guide sequences suitable for pan‐HLA‐A and pan‐HLA‐C knockout, respectively. Pan‐HLA‐B targeting was not pursued due to the lack of a suitable HLA‐B antibody for flow cytometry analysis. We randomly selected and cloned two A*02:01‐specific and four A*03:01‐specific sgRNAs, along with a non‐targeting control, into the LentiCRISPRv2 vector. These constructs were used to generate stable knockout cell lines based on the SK‐RC‐01 cells. Flow cytometry analysis showed that both A*02:01‐targeting sgRNAs resulted in near‐complete loss of A*02:01 protein surface expression, whereas only two of the four A*03:01‐targeting sgRNAs produced a significant knockout effect (Figure 4). To assess allele specificity, we evaluated potential cross‐reactivity among these sgRNAs. None of them demonstrated significant off‐target effects (Figure 4). We also generated stable cell lines for pan‐HLA‐A or pan‐HLA‐C knockout. Among these, two out of four clones for each target showed almost complete depletion of pan‐HLA‐A or pan‐HLA‐C expression, respectively (data not shown). The expression of other HLA genes were unaffected in the knockout cells analysed (Figure 5).

### 3.4 Suppression of HLA‐Restricted T‐Cell Activation by Allele‐Specific HLA Knockout

**FIGURE 6:**
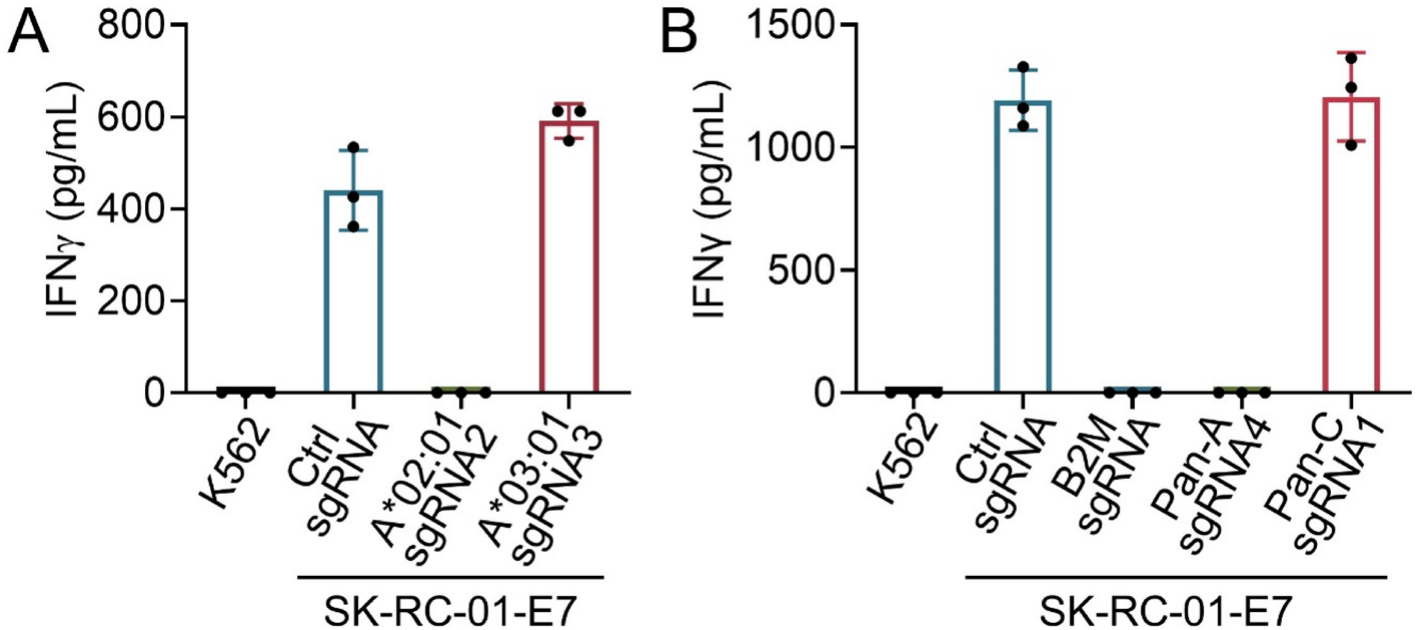
Functional validation of allele‐specific HLA knockout. (A, B) IFN‐γ secretion measured by ELISA after co‐culture of HPV16 E7‐specific TCR‐T cells with SK‐RC‐01‐E7 cells bearing the indicated HLA gene knockouts. Co‐culture with K562 cells was included as a negative control.

To functionally validate the allele‐specific HLA knockouts, we overexpressed HPV16‐E7 protein in SK‐RC‐01 cells and generated knockouts for HLA‐A*02:01, HLA‐A*03:01, pan‐HLA‐A and pan‐HLA‐C using the sgRNA previously validated. These modified cells were then co‐cultured with human T cells transduced with a TCR specific for the HLA‐A*02:01‐restricted HPV16 E7_11‐19_ epitope [18]. TCR‐T cells can be activated by HLA‐A*02:01 ^+^ SK‐RC‐01 cells with overexpression of HPV16‐E7, as measured by interferon‐γ (IFN‐γ) secretion. However, IFN‐γ secretion was abolished in HLA‐A*02:01 and pan‐HLA‐A knockout cells, but not in HLA‐A*03:01 or pan‐HLA‐C knockouts (Figure 6A,B), confirming the allele specificity and functional relevance of the allele‐specific HLA Class I knockout.

## 4 Conclusion

HLA‐Knockout is a user‐friendly web tool designed to facilitate the selection of guide sequences for allele‐specific knockout of HLA Class I genes using the spCas9 system. Although numerous tools exist for sgRNA design, none are optimised for targeting HLA Class I alleles due to their extensive polymorphism. HLA‐Knockout leverages the IPD‐IMGT/HLA Database to enable customised selection of both target and non‐target alleles, ensuring high specificity. We have experimentally validated that the guide sequences identified by HLA‐Knockout can efficiently and selectively knock out the intended alleles, preserve expression of non‐targeted alleles and abolish antigen‐specific T‐cell activation restricted by the targeted allele. This tool will be valuable for studies involving autoimmune diseases, organ transplantation and cancer immunology. The current version is only applicable to canonical HLA Class I gene alleles that are fully annotated in the IPD‐IMGT/HLA database. Future updates will incorporate custom sequence input, support for Class II HLA genes, additional Cas9‐based systems beyond spCas9 and genome‐wide off‐target prediction outside the HLA locus.

## Author Contributions

J.C. and Q.X. conceived and initiated the project and oversaw the overall study design. C.M. developed the tool, built the website, performed simulation analyses and contributed to manuscript preparation. S.W. performed most experiments with assistance from L.J. J.C. and Q.X. interpreted the data and co‐wrote the manuscript.

## Funding

This work was supported by Rutgers Cancer Institute of New Jersey (P30CA072720), Nanjing University, Science Fund Program for Distinguished Young Scholars (Overseas), National Natural Science Foundation of China Grant (82473308), and Fundamental Research Funds for the Central Universities (2025300355).

## Conflicts of Interest

The authors declare no conflicts of interest.

## Data Availability

The HLA‐Knockout server is available at https://hlaknockout.rutgers.edu. The source code for HLA‐Knockout has been deposited on GitHub and is publicly available at https://github.com/Cao‐Lab‐Rutgers/HLA_knockout. Source data for all figures (simulation results, raw flow cytometry files and gating templates, and ELISA measurement data) are available from the corresponding author upon reasonable request.
